# Characterization of Genes for Beef Marbling Based on Applying Gene Coexpression Network

**DOI:** 10.1155/2014/708562

**Published:** 2014-01-30

**Authors:** Dajeong Lim, Nam-Kuk Kim, Seung-Hwan Lee, Hye-Sun Park, Yong-Min Cho, Han-Ha Chai, Heebal Kim

**Affiliations:** ^1^Division of Animal Genomics and Bioinformatics, National Institute of Animal Science, Rural Development Administration, Suwon 441-706, Republic of Korea; ^2^National Agricultural Products Quality Management Service (NAQS), Seoul 150-804, Republic of Korea; ^3^Department of Food and Animal Biotechnology, Seoul National University, Seoul 151-742, Republic of Korea

## Abstract

Marbling is an important trait in characterization beef quality and a major factor for determining the price of beef in the Korean beef market. In particular, marbling is a complex trait and needs a system-level approach for identifying candidate genes related to the trait. To find the candidate gene associated with marbling, we used a weighted gene coexpression network analysis from the expression value of bovine genes. Hub genes were identified; they were topologically centered with large degree and BC values in the global network. We performed gene expression analysis to detect candidate genes in *M. longissimus* with divergent marbling phenotype (marbling scores 2 to 7) using qRT-PCR. The results demonstrate that transmembrane protein 60 (TMEM60) and dihydropyrimidine dehydrogenase (DPYD) are associated with increasing marbling fat. We suggest that the network-based approach in livestock may be an important method for analyzing the complex effects of candidate genes associated with complex traits like marbling or tenderness.

## 1. Introduction

Marbling (intramuscular fat) is a major trait in characterzing beef quality and an important factor for determining the price of beef in the Korean beef market. It is also a complex trait, which is obtained from many genes like tenderness. Therefore, a complex trait like marbling demands such an approach, because no single factor determines a large proportion of the trait variations in the population [1]. For this reason, systems biology approach has been useful to identify genes that underlie complex trait from network of genetic interactions among all possible genes. Furthermore, patterns of covariation in the expression of multiple loci can be used to build networks that show relationships between genes and between genes and functional traits. These networks provide information on the genetic control of complex traits and can help identify causal genes that affect gene function rather than gene expression [2]. System-oriented approaches have been applied by animal geneticists to investigate livestock traits [3–5], resulting in the identification and characterization of economically important causal transacting genes within QTL regions. These *trans*-QTL regions share a common biological function (e.g., similar gene ontology function, metabolic pathway, and transcriptional coregulation) [6–8]. In the case of bovines, several countries identify quality challenges, such as marbling, meat tenderness, carcass weight, muscling, and fat cover. Three genes were identified as being significantly correlated with bovine skeletal muscle based on microarray data from a gene network [9]. Jiang et al. [10] reported that the genetic network was associated with 19 economically important beef traits. This report suggested the three candidate gene approach as targets. Therefore, we need a systemic approach in order to identify candidate genes in the network analysis among many genes related to marbling within QTL intervals. A gene coexpression network (GCN) is a gene correlation network created from expression profiling, with each gene having several neighbors, and is useful for identifying genes that control quantitative phenotypes.

In this study, we introduce a systemic approach involving network analysis of marbling score-related genes and experimental evidence confirming that highly connected genes (hubs) are significantly different between high- and low-marbling groups.

## 2. Materials and Methods 

Our analysis involved three main steps: (1) finding candidate genes in the Animal QTL database and analyzing the results of microarray experiments from the Gene Expression Omnibus (GEO) database, (2) constructing coexpression networks related to the “marbling score” trait and analyzing the network topology and functional enrichment, and (3) investigating gene expression for hub genes using quantitative reverse-transcription PCR (qRT-PCR).

### 2.1. Identification of Candidate Genes Associated with the Marbling Score

To determine candidate genes associated with the marbling score within QTL intervals, we obtained genomic positions of the “marbling score” trait using “QTL location by bp” information from the Animal QTL database (http://www.animalgenome.org/cgi-bin/QTLdb/BT/index). Most of QTLs are identified in the different regions in a chromosome. There are rare regions of overlap. Therefore, we select the genes associated with marbling score from Animal QTL database with QTL IDs that have marker information in term of “marbling score” within Animal Trait Ontology (ATO) category. In the GEO database (http://www.ncbi.nlm.nih.gov/geo/), all data from microarray experiments related to bovines were used: GEO series (GSE) 15544, GSE 15342, GSE 13725, GSE 6918, GSE 10695, GSE 12327, GSE 9256, GSE 12688, GSE 11495, GSE 11312, GSE 7360, and GSE 9344. Table S1 (see Table S1 in Supplementary Material available online at http://dx.doi.org/10.1155/2014/708562 shows the summary of microarray data sets [11–20]. All arrays were processed to determine the robust multiarray average (RMA) [21] using the “affy” software package [22]. Expression values were computed in detail from raw CEL files by applying the RMA model of probe-specific correction for perfect-match probes. These corrected probe values were then subjected to quantile normalization, and a median polish was applied to compute one expression measure from all probe values. Figure S1 shows the distribution before and after normalization. Resulting RMA expression values were log2-transformed. We determined mean intensity for an expression intensity of each gene matching to at least two probes. Finally, we used 844 probes among 1,260 redundant probes associated with marbling for network construction.

### 2.2. Gene Coexpression Network Construction and Network Module Identification

In coexpression networks, we refer to nodes as genes whose degrees indicate the number of links connected by the node. We extracted expression values for 844 genes and evaluated pairwise correlations between the gene expression profiles of each pair of genes using Pearson's correlation coefficients (denoted as *r*). The unweighted network encoded gene coexpression as binary information (connected = 1, unconnected = 0) using a “hard” threshold. In contrast, the weighted network represented “soft” thresholding that weighed each connection as a continuous number [0, 1]. The power adjacency function *a*_*ij*_ = |cor(*x*_*i*_, *x*_*j*_)|^*β*^ was used to construct a weighted network as the connection strength between two genes. We investigated soft thresholding with the power adjacency function and selected a power of beta (*β*) = 7. A major aim of coexpression network analysis is to determine subsets of nodes (modules) that are tightly connected to each other. To organize genes into modules, we used a module identification method based on a topological overlap dissimilarity measure [23] in conjunction with a clustering method, which detected biologically meaningful modules. The topological overlap of two nodes refers to their relative interconnectedness. The topological overlap matrix (TOM) *Ω* = [*ω*_*ij*_] provides a similarity measure, which has proven useful in biological networks [24], where *l*_*ij*_ = ∑_*u*_ *a*_*iu*_ *a*_*uj*_ and *k*_*i*_ = ∑_*u*_ *a*_*iu*_ is the node connectivity as follows:

(1) ωij=lij+aijmin⁡⁡{ki,kj}+1−aij.

In the case of our network, *l*_*ij*_ equals the number of nodes to which both *i* and *j* are connected. To identify modules, we used TOM-based dissimilarity *d*_*ij*_^*w*^ (*d*_*ij*_^*w*^ = 1 − *ω*_*ij*_) in a hierarchical cluster analysis. Each module represents a group of genes with similar expression profiles across the samples and the expression profile pattern is distinct from those of other modules. The weighted gene coexpression analysis (WGCNA) software packages for R were used to identify coexpression values associated with marbling score [25].

To characterize the overall network topology, we used node degree (or connectivity), betweenness centrality (BC) [1]. The degree of a node is the number of connections or edges the node has with other nodes. The degree distribution of a network has a generalized power-law form *p*(*k*) ~ *k* ^−*r*^, which is the defining property of a scale-free network [26]. The genes of highly connected nodes to nodes with few connections (hubs) play an important role as a local property in a network [27]. A node with high BC has great influence over what flows in the network; BC may play a major role as a global property since it is a useful indicator for detecting bottlenecks in a network. For node *k*, BC is the fraction of the number of shortest paths that pass through each node [28] and is defined as

(2) b(k)=∑i,jbi→j(k)=∑i,jgi→jkgi→j,

where *g*_*i*→*j*_ is the number of the shortest geodesic paths from node *i* to node *j* and *g*_*i*→*j*_^*k*^ is the number of geodesic paths among *g*_*i*→*j*_ from node *i* to node *j* that pass through node *k*. We calculated BC as global properties according to all nodes in a network. From the results of the network topology analysis, we selected high-degree nodes and high-centrality nodes as key drivers that are most associated with our trait of interest in the network.

### 2.3. Functional Enrichment Analysis

We performed functional enrichment analysis against the 844 genes that were associated with marbling score enrichment in the Gene Ontology and KEGG pathway terms using the database for Annotation, Visualization, and Integrated Discovery (DAVID) tool (http://david.abcc.ncifcrf.gov/). Each module was also analyzed separately, regardless of whether the gene module was significantly enriched with known ontology or pathway terms. The software calculates a Fisher's exact test *P*-value and provides a corrected *P*-value to avoid multiple test issues.

### 2.4. Confirmation of Gene Expression Results by Quantitative Reverse-Transcription PCR (qRT-PCR)

We determined whether any associations existed between expression levels and intramuscular fat content in *M. longissimus* tissue in Korean cattle (Hanwoo). All experimental procedures and care of animals were conducted in accordance with the guidelines of the Animal Care and Use Committee of the National Institute of Animal Science in Korea. Twelve steers from each of low-marbled group (9.54 ± 1.35%) and high-marbled group (20.84 ± 1.52%) were used in this study for real-time PCR and statistical analyses (Table 1). Total RNA was prepared from each tissue sample (100 mg) with TRIzol reagent (Invitrogen Life Technologies, Carlsbad, CA, USA) and purified using an RNeasy MinElute Clean-up Kit (Qiagen, Valencia, CA, USA). RNA concentration was measured with a NanoDrop ND-1000 spectrophotometer (Thermo Scientific, Waltham, MA, USA). RNA purity (*A*_260_/*A*_280_) was over 1.90. For cDNA synthesis, 2 *μ*g RNA was reverse transcribed in a 20 *μ*L reaction volume using random primers (Promega, Madison, WI, USA) and reverse transcriptase (SuperScript II Reverse Transcriptase; Invitrogen Life Technologies). Reactions were incubated at 65°C for 5 min, 42°C for 50 min, and then at 70°C for 15 min to inactivate the reverse transcriptase. Real-time PCR was performed using a 2× Power SYBR Green PCR Master mix (Applied Biosystems, Foster City, CA, USA) with a 7500 real-time PCR system (Applied Biosystems) using 10 pM of each primer. PCR was run for 2 min at 50°C and 10 min at 95°C, followed by 40 cycles at 95°C for 10 s and then at 60°C for 1 min. Following amplification, a melting curve analysis was performed to verify the specificity of the reactions. The endpoint used in the real-time PCR quantification, Ct, was defined as the PCR threshold cycle number. We selected 11 hub genes (6 genes with large degree and 5 with large BC) from the network topology analysis. To determine major patterns in the 11 gene expression data, we performed principal component analysis (PCA) for the nodes with large degree and BC. A regression model was used to examine the association between gene expression value and intramuscular fat content using the “lm” function in R. This produced the following equation:

(3) IMFij=μ+Expressioni+Ageij+Residualij,

where expression is a normalized gene expression value, *μ* is an overall mean, IMF_*ij*_ is the intramuscular fat content of each animal from gene *i*  (*i* = 1,…, 11) and animal *j*  (*j* = 1,…, 12), and Age_*ij*_ is slaughtering age in months, which was included as a covariate; the mRNA level of the *β*-actin, ribosomal protein, large, P0 (RPLP0) gene was also introduced as a covariate [29].

## 3. Results and Discussion

### 3.1. Identification of the Global Coexpression Network

The nodes represent candidate genes obtained from the animal QTL database and microarray data, and the links between the nodes represent the association between expression profiles across all microarray samples. The absolute value of Pearson's correlation coefficient was calculated for all pairwise comparisons.

**Figure 1:**
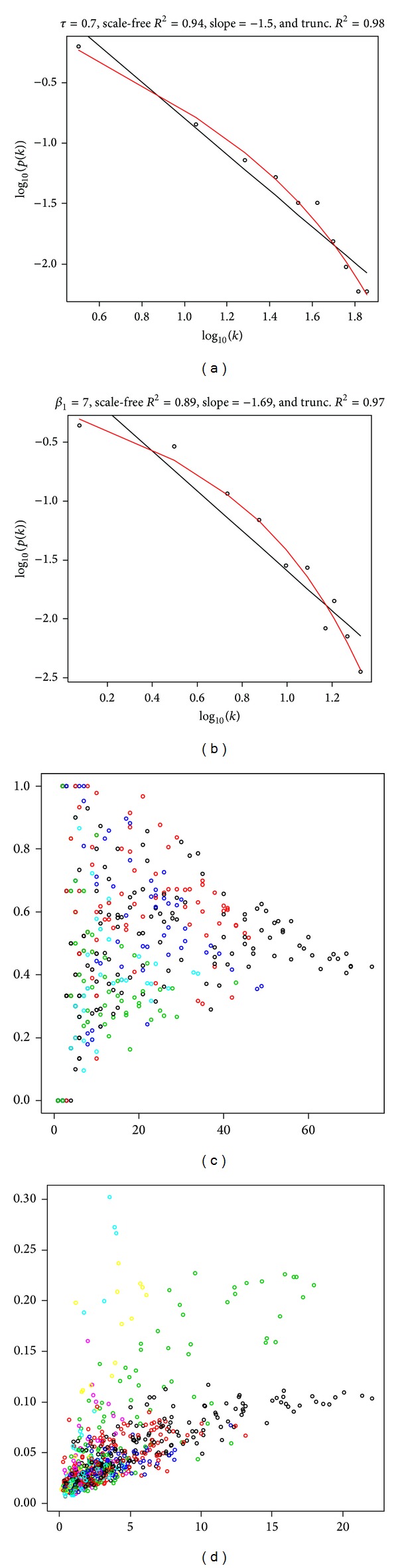
Comparison of weighted and unweighted networks associated with marbling score. (a) The scale-free plot for unweighted network (*τ* = 0.7). (b) The scale-free plot for weighted network (*β* = 7). Two types of network approximately follow power-law distribution. (c) The scatter plot of clustering coefficient (*y*-axis) and connectivity (*x*-axis) in unweighted network. Genes are colored by module membership. (d) The scatter plot of clustering coefficient (*y*-axis) and connectivity (*x*-axis) in weighted network.

We constructed a weighted gene coexpression network associated with the marbling score using soft threshold. A comparison with the weighted and unweighted gene coexpression network is required before decision making. This correlation matrix was transformed into a matrix of adjacency using a “hard” threshold (*τ*, 0.7) and a “soft” threshold (*β*, 7.0), producing a gene coexpression network. The network follows a power-law (*P*(*k*) ~ *k* ^−*r*^) degree distribution, where *r* is the degree exponent and ~ indicates “proportional to.” We examined whether the coexpression network followed a power-law distribution with an exponent of approximately −1.8 [30] using log⁡(*p*(*k*)) and log⁡(*k*), that is, the model fitting index, *R* ^2^ of the linear module that regresses log⁡(*p*(*k*)) and log⁡(*k*). Figures 1(a) and 1(b) show a scale-free topology plot of the network constructed with the power adjacency function. This plot between log⁡_10_(*p*(*k*)) and log⁡_10_(*k*)*k* shows that the network approximately follows a scale-free topology (black regression line, *R* ^2^ = 0.94 in the unweighted network and, *R* ^2^ = 0.89 in the weighted network). We also found that the connectivity distribution *p*(*k*) was better modeled using an exponentially truncated power-law (*k*) ~ *k* ^−*r*^exp⁡⁡(−*αk*), where *R* ^2^ = 0.98 in the unweighted network and *R* ^2^ = 0.97 in the weighted network [31]. Thus, our network has characteristics of a scale-free network whose degree distribution approximates a power law.

We also examined the relationship between the clustering coefficient and the connectivity of each gene. The clustering coefficient (CC) is an indicator of network structure, which quantifies network modularity and how close the node and its neighbors are. We observed an inverse relationship or a triangular region between the clustering coefficient and connectivity in the unweighted network (Figure 1(c)). The decrease in the clustering coefficient indicates overlap between modules. This is consistent with results reported by previous researchers [18, 31]. However, the result may be an artifact of hard thresholding [32]. In contrast to the unweighted network, the weighted network showed a positive correlation between connectivity and the cluster coefficient in most modules and across modules, the clustering coefficient showed considerable variation (Figure 1(d)). This relationship is shown in the weighted network analysis; for highly connected nodes in a module, the corresponding correlation matrix is roughly factorizable [32]. The unweighted network has the advantage of a strong correlation pattern between genes, which may lead to erroneous estimates or false positives. The grey modules included 359 (unweighted) and 76 (weighted) genes that we were not able to analyze in our study because the modules were not clustered. In the unweighted network, the adjacency matrix encodes whether a pair of nodes is connected. Therefore, the hard threshold may cause a loss of information and sensitivity because of the choice of threshold and artifact from clustering coefficient result. For these reasons, we found that the results of the weighted network analysis were highly robust to the selection of the soft parameter *β* when it was used for module identification, connectivity definition.

Most biological networks are characterized by a small number of highly connected nodes, while most of the other nodes have few connections [28]. The highly connected nodes act as hubs that mediate interactions between other nodes in the network. In the whole network and the weighted network, the network topology information of the hub candidates is summarized in Table 2. BC is an indicator of a global central node. The effect of removing nodes with large BC values is similar to that of removing hub nodes because large BC nodes are correlated with hub nodes [33]. However, large BC nodes are not hub nodes; they imply that a site is relatively central between all other sites. This means that such sites are advantageously located to act as intermediaries. Therefore, we investigated communication between nodes and confirmed that hub and large BC nodes are located in the topological center of the network by calculating BC for the whole network. Degree and BC determine if hubs have local or global importance in the network, respectively. For example, transmembrane protein 60 (TMEM60), maelstrom (MAEL), and histidine triad nucleotide binding protein 1 (HINT1) are hub nodes that have large degrees and large BC values throughout the entire network. However, dihydropyrimidine dehydrogenase (DPYD) and ELOVL fatty acid elongase 4 (ELOVL4) are near the global center of the network with large BC values (Table 2). Further, we investigated gene expression with large degree and BC to find candidate genes associated with marbling score.

### 3.2. Detection of Coexpression Gene Modules Related to the Marbling Score

**Figure 2:**
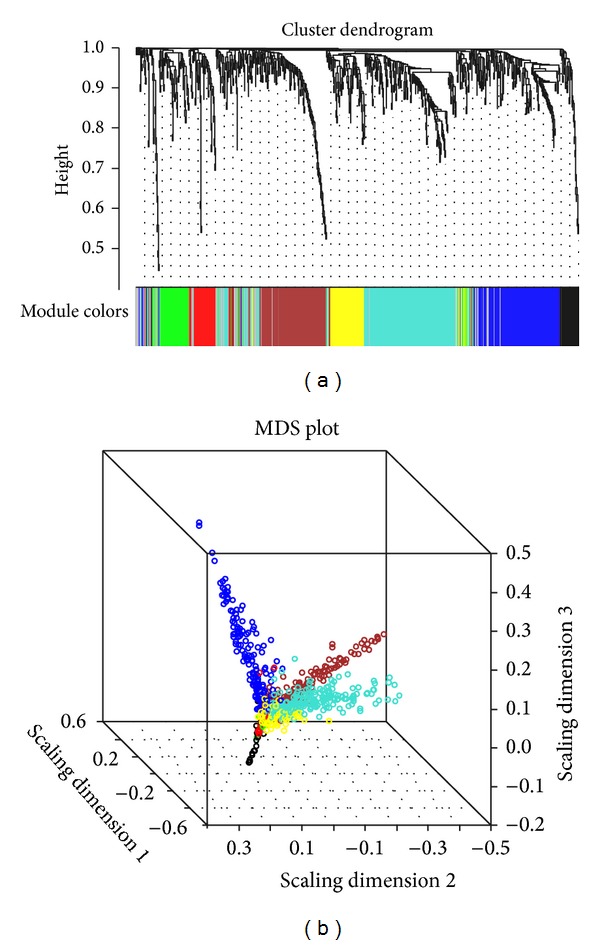
(a) Hierarchical clustering of marbling score-related genes and visualization of gene modules. The colored bars (below) one directly consistent with the module (color) for the clusters of genes. Distance between genes is shown as height on the *y*-axis. (b) Multidimensional scaling plot of the weighted network. Genes are represented by a dot and colored by module membership. The distance between each gene is indicated by their topological overlap. This representation explains how the module is related to the rest of the network and how closely two modules are linked.

To find clusters (gene modules) of highly correlated genes, we used average linkage hierarchical clustering, which uses TOM as dissimilarity. We choose a height cutoff of 0.99 to identify modules using a dynamic cut-tree algorithm. Connectivity is the number of nearest neighbors of a node and the effective mean degree is the average degree of all nodes except isolated nodes. We are able to identify seven distinct modules (except for the “grey” module, which is not grouped into any module) for groups of genes with high topological overlap: turquoise, black, yellow, brown, blue, green, and red.  Figure 2 shows the visualization of the modules in the weighted network. It consisted of ranges of gene modules from 38 (black) to 219 genes (turquoise), and mean overall connectivity ranged from 1.92 (black) to 5.77 (turquoise).

Gene modules are important for identifying genes related to the trait of interest because they may be highly correlated in biological pathways. Each module was analyzed through functional enrichment analysis using gene ontology or KEGG pathway terms to understand the biological significance of the module genes and to determine putative pathways. The seven modules and their representative pathway terms were turquoise, other glycan degradations (bta00511, *P*-value = 0.01); yellow, oxidative phosphorylation (bta00190, *P*-value = 0.009); blue, hematopoietic cell lineage (bta04640, *P*-value = 0.006); brown, PPAR signaling pathway (bta03320, *P*-value = 0.04); green, dilated cardiomyopathy (bta05414, *P*-value = 0.04); red, natural killer cell-mediated cytotoxicity (bta04650, *P*-value = 0.0007); and black, no significant term. Marbling (intramuscular fat)-related genes have been identified which are directly involved in lipid and fatty acid metabolism. These genes are not independently associated with marbling but interact in functionally important pathways [26] such as the peroxisome proliferator-activated receptors (PPAR) signaling pathway, adipocyte differentiation, lipid accumulation, and adipogenesis. We also found that the brown module has significant GO terms related to the marbling trait, the lipid biosynthetic process (GO:0008610, *P* = 0.002), and the lipid metabolic process (GO:0006629, *P* = 0.004). The lipid biosynthetic process involved the following genes: TECR, PMVK, LASS4, HMGCS2, APOA2, MGST2, FDFT1, and FDPS. The lipid metabolic process included the following genes: CROT, PI4KB, LASS4, HMGCS2, APOA2, HPGD, MGST2, FDFT1, and FDPS. Investigations on lipid metabolism in harvested animals have centered on research into adipose tissues [34, 35]. Therefore, we focused on the brown module prior to gene ontology and the pathway analysis and performed a module-based analysis. For the brown module genes, intramodular connectivities were calculated because they are relatively robust with respect to the whole network and more biologically meaningful than the whole network. Retinoic acid receptor-related orphan receptor c (RORC) had a large degree in both the whole network and the blue module. RORC is significantly associated with intramuscular fat, marbling score [36], and fatness [37]. In beef cattle, adipose tissue formation is associated with genetic background, development, and biological pathways. PPAR*γ*, CCAAT-enhancer binding proteins (CEBP*α*, CEBP*β*), and sterol regulatory element binding proteins (SREBP 1c) are reportedly directly or indirectly related to the regulation of adipogenesis [38]. PPAR*γ* is known as a master regulator of adipogenesis [39]. We found genes associated with the PPAR*γ* signaling pathway in the brown module, that is, APOA2, ANGPTL4, FABP5, and ACSL6. APOA2, ANGTPTL4, and ACSL6 are involved in lipid metabolism. ANGPTL4 is a well-known PPAR target gene and has multiple metabolic effects such as glucose and lipid metabolism [40]. Moreover, its expression is increased by PPAR*γ* activation both *in vitro* and *in vivo* [41]. Fatty acid-binding proteins (FABP4 or FABP5) are candidate genes for the marbling (intramuscular fat deposition) trait; they interact with peroxisome proliferator-activated receptors and bind to hormone-sensitive lipase, therefore playing an important role in lipid metabolism and glucose homeostasis in adipocytes [42, 43]. ACSL6 is a member of the ACSL isoforms [44], which activates fatty acids of varying chain lengths and is an insulin-regulated gene [45]. It is directly involved with fatty acids in diverse metabolic pathways of lipid synthesis [46]. We examined commonly linked edges (genes) against the genes involved in PPAR signaling pathway in the brown module of weighted network. The following genes are connected to PPAR signaling pathway related genes (APOA2, FABP5, and ANGPTL4): ILVBL, APCS, CREB3L3, ANXA13, CHIA, LRG1, HAO2, ALDH9A1, HMGCS2, TUBB4, HNF4G, and GSTM1.

### 3.3. Confirmation of Gene Expression Results by Quantitative Reverse-Transcription PCR (qRT-PCR)

To further confirm gene expressions and relationships, 11 genes (6 genes with large degree and 5 with large BC) were selected after network topology analysis. Then, we conducted experimental validation of whether large degree and large BC nodes were related to marbling (intramuscular fat). We investigated the expression levels of eleven candidate genes in *M. longissimus* muscle between two distinct intramuscular fat content groups. Marbling is highly correlated with IMF content with phenotype in the previous reports [47, 48]. Our data shows that correlation coefficient between marbling and IMF content is highly correlated (*r* = 0.81, *P*-value = 0.0013). The Pearson's correlation coefficients of marbling and two gene's expression levels are highly correlated and also statistically significant by regression analysis (TMEM60: *r* = 0.72, *P*-value = 0.013, DPYD: *r* = 0.85, and *P*-value = 0.001). Therefore, we identified candidate genes associated with marbling and then confirmed candidate genes in IMF phenotype.

**Figure 3:**
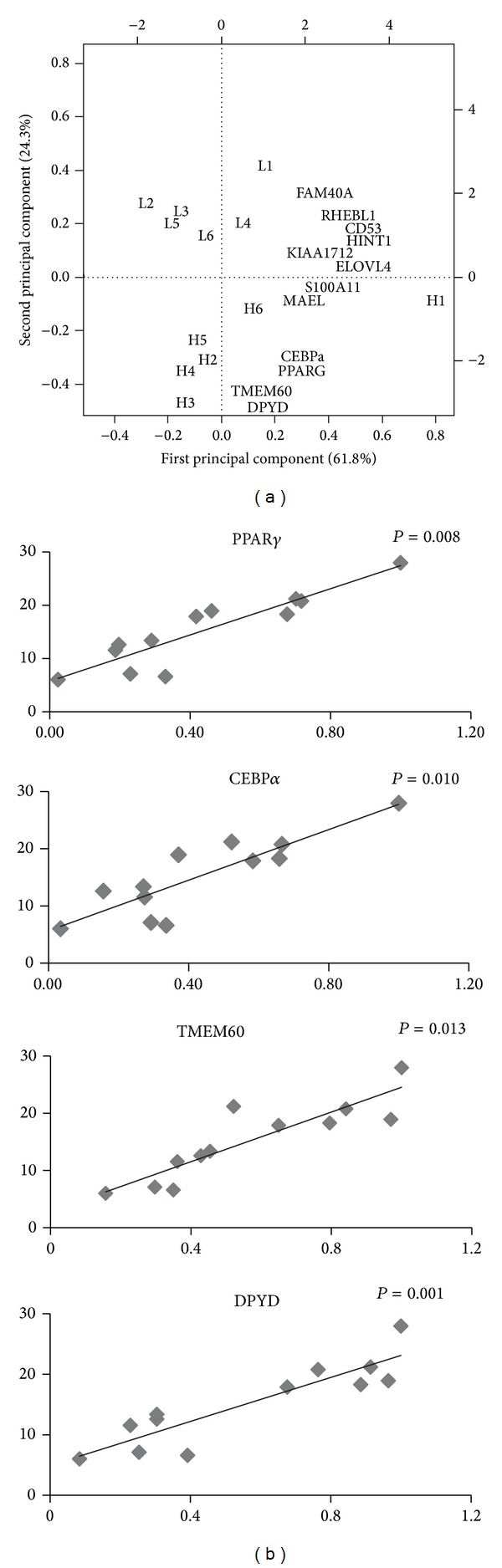
Analysis results of gene expression data by regression model and PCA. (a) Biplot of the first two principal components. The symbols of L (left) and H (right) represent low- and high-marbled samples in the plot, respectively. (b) Regression analysis between expression level (*x*-axis) and intramuscular fat content (%, *y*-axis) for each sample. CEBPa and PPARG were used as indicators of marbling (intramuscular fat).

First, we investigated the expression levels of two genes (Figure 2(b)), PPAR*γ* and CEBP*α*, as indicators of fat accumulation, which are the major transcription factors regulating adipogenesis [49]. The mRNA expression levels of PPAR*γ* and CEBP*α* were more highly expressed in the high-marbled group (*P* ≤ 0.01), In the present study, we identified two genes, TMEM60 and DPYD, which were approximately 2.1 and 3.2 times higher in the high-marbled group and also upregulated with intramuscular fat content increases (*P* < 0.05) (Table 3 and Figure 3(b)). These genes have not been reported to be associated with marbling. TMEM60 plays an important role as a hub node in both the whole network and the gene module (turquoise). It participates in a wide range of biological functions related to marbling in the global network. TMEM60 belongs to a family of membrane proteins of unknown function and has three domains, two of which have unknown functions (PF06912 and PF12036) and transmembrane Fragile-X-F protein (PF10269). TMEM60 might be associated with Fragile-X syndrome, which results in low muscle tone and tension or resistance to movement in a muscle. Transmembrane protein might be affected by a wide range of biological mechanisms, such as body composition and insulin action. It is known to be expressed abundantly in preadipocyte. Transmembrane protein 182 (TMEM182) is upregulated during the myoblasts to myotubes in the adipocyte and muscle lineage [50]. More detailed studies of muscle and fatty acids profiles of bovine marbling trait are necessary to evaluate this possibility. DPYD is involved in our module of interest (brown) and is not a hub node in either the whole network or in the brown module. However, it plays an important role in communication and connections between genes that are linked to functions or pathways associated with the marbling trait, acting like a bridge. DPYD is associated with severe fluoropyrimidines (FP) toxicity and is known to be involved in FP-treated cancer patients. We determined that the genes connected to DPYD are involved in the nitrogen compound metabolic process (GO:0006807), oxidation reduction (GO:0055114), the cellular biosynthetic process (GO:0044249), the biosynthetic process (GO:0009058), the cell cycle (GO:0007049), and the primary metabolic process (GO:0044238) from functional enrichment analysis. Nitrogen metabolism is associated with the ability of the rumen and has an important role of formation of amino acids in beef steer [51]. It is also known to have a strong influence on lipid metabolism and fatty acids metabolism [52]. Moreover, most of these genes function is involved in the oxidation reduction process for transport of energy. Zhao et al. [38] reported that the differentially expressed genes related to fat accumulation were shown to have function of oxidation reduction process.

PCA is a useful tool for data simplification and visualization of relationships. Therefore, we applied PCA to the 11-gene expression data set.  Figure 3(a) showed that the relationships among these genes were illustrated by PCA. The first two principal components explained approximately 86.1% of the total variance, allowing most of the information to be visualized in two dimensions. The analysis indicated that the most important pattern of gene expression (PC1, accounting for 61.8% of variance in the data) was associated with differences in intramuscular fat. Individual samples were clearly partitioned into two separate groups, high- and low-marbled groups based on PC2. In this analysis, the second PC illustrated the link among HINT1, KIAA1712, RHEBL1, FAM40A, CD53, ELOVL4, and CTSS genes, which have a positive relationship with PC2. On the other hand, PPAR*γ*, CEBP*α*, MAEL, TMEM60, S100A11, and DPYD genes have a negative relationship with PC2. Our experimental results suggest that these genes warrant further investigation as metabolic indicators of marbling.

## 4. Conclusion

In this study, we extracted gene list related to the marbling score trait from the Animal QTL database and microarray experiments from the GEO database. We subsequently constructed a global network and a weighted gene coexpression network based on Pearson's correlation matrix that displayed degrees using a power-law distribution, with an exponent of approximately −2. Hub genes were identified; they were topologically centered with large degree and BC values in the global network. Moreover, they were significantly correlated with three (turquoise, red, and brown) gene modules. Finally, we confirmed that the expressions of hub (TMEM60) and nodes with large BC values (DPYD) were consistent with the network topology analysis. These genes have not been reported previously in bovine gene expression studies on marbling. Further studies should be conducted to identify biological mechanism of the genes in the network associated with bovine marbling.

## Supplementary Material

Table S1 shows “Summary of microarray data sets”.
Figure S1 shows “the distribution before and after normalization”.

## Figures and Tables

**Table 1:** Summary statistics of tissue sample for gene expression analysis.

Group	Animal	Age (month)	IMF (%)	Group	Animal	Age (month)	IMF (%)
Low	509	26	7.11	High	508	26	27.97
	537	27	6.02		582	31	18.94
	539	26	11.56		603	31	18.3
	543	27	6.6		648	29	20.78
	590	27	12.6		652	29	17.89
	706	28	13.37		685	29	21.2

**Table 2:** The network topology information of the hub gene in the weighted network and the global network.

Gene name	The weighted network			The global network		
	Module	Correlation	*P* value	Degree	Betweenness centrality (BC)	Closeness centrality (CC)
MAEL	Turquoise	0.32	3.14*E* − 87	76	0.0150347	0.3216747
HINT1	Turquoise	−0.37	1.20*E* − 70	74	0.0161593	0.3211731
KIAA1712	Turquoise	0.37	2.39*E* − 72	73	0.0091218	0.3205068
TMEM60	Turquoise	0.23	2.70*E* − 54	68	0.0177178	0.3172161
RHEBL1	Turquoise	0.21	1.41*E* − 66	67	0.0174118	0.3143118
FAM40A	Turquoise	0.19	4.76*E* − 59	67	0.0138916	0.3173791
S100A11	Turquoise	−0.59	2.35*E* − 13	20	0.0425473	0.2635126
CD53	Red	0.17	2.87*E* − 44	12	0.0404111	0.232482
DPYD	Brown	0.13	7.65*E* − 37	42	0.0403153	0.312405
ELOVL4	Turquoise	0.17	1.62*E* − 20	10	0.0377276	0.2584429
CTSS	Red	−0.65	3.49*E* − 20	7	0.0366287	0.2780995

**Table 3:** Gene network and expression analysis of genes with large degree and BC. We selected 11 hub genes (6 genes with large degree and 5 with large BC) from the network topology analysis and confirmed gene expression for Hanwoo marbling using qRT-PCR.

Gene network^a^	Gene	Full name		Expression^b^		Relationship^c^	*P* value^d^
				Low	High		
Large degree	MAEL	Maelstrom homolog	Turquoise	0.29	0.33	Positive	0.871
	HINT1	Histidine triad nucleotide binding Protein 1	Turquoise	0.41	0.25	Negative	0.118
	KIAA1712	KIAA1712	Turquoise	0.34	0.21	Negative	0.283
	TMEM60	Transmembrane protein 60	Turquoise	0.34	0.76	Positive	**0.013**
	RHEBL1	Ras homolog enriched in brain-like 1	Turquoise	0.45	0.31	Negative	0.544
	FAM40A	Hypothetical protein LOC511120	Turquoise	0.54	0.30	Negative	0.528

Large BC	S100A11	S100 calcium binding protein A11	Turquoise	0.34	0.36	Positive	0.616
	CD53	CD53 molecule	Red	0.41	0.28	Negative	0.901
	DPYD	Dihydropyrimidine dehydrogenase	Brown	0.26	0.84	Positive	**0.001**
	ELOVL4	Elongation of very long chain fatty acid-like 4	Turquoise	0.38	0.33	Negative	0.991
	CTSS	Cathepsin S	Red	0.33	0.23	Negative	0.765

^a^Expression and promotor binding indicate that the regulator changes the expression level and binds the promoter of the target.

^b^Expression showed means of normalized expression value of each gene within low- and high-marbled groups.

^c^Relationship indicated expression relationship of each gene against the intramuscular fat from PCA analysis.

^d^ *P*-value was calculated by the regression analysis.

The bold type indicates significant differences at *P* ≤ 0.05 between high and low-marbled groups.
