# Supplementary material for: Characterization of Genes for Beef Marbling Based on Applying Gene Coexpression Network
Source: Int J Genomics. 2014 Jan 30;2014:708562. doi: 10.1155/2014/708562 (PMC3929194; doi:10.1155/2014/708562)
Supplement: Supplementary file 1 — Table S1 shows “Summary of microarray data sets”. Figure S1 shows “the distribution before and after normalization”. [file 708562.f1.zip › 708562.f1/mat.708562.v1.docx]

**Supplementary Data 1. Primer sequences for real-time PCR**

| Gene symbol | Full name | Primer sequence (5`-3`) | | GenBank Acc. No. |
| --- | --- | --- | --- | --- |
| MAEL | maelstrom homolog | Forward | ACACAAGGTGCAAGTGGCATGAAG | NM_001038193.1 |
|  |  | Reverse | ACATGAGCCTCTGTGAGCTGGATT |  |
| HINT1 | histidine triad nucleotide binding protein 1 | Forward | TGGGCCTGAAGAAAGGCTATCGAA | NM_175812.2 |
|  |  | Reverse | TTCATCTGCCGACCTCCAAGAACA |  |
| KIAA1712 | KIAA1712 | Forward | TTTGAGAGGAGCAGCATTCCCAGA | NM_001076833.1 |
|  |  | Reverse | CGTTTCCTACTTGGCAGGCAGATT |  |
| TMEM60 | transmembrane protein 60 | Forward | AACTGGATGAGAAGGCACCTTGGA | NM_001076988.1 |
|  |  | Reverse | TGTGTGATCCATGTCGAGGGTCAA |  |
| RHEBL1 | Ras homolog enriched in brain like 1 | Forward | AGCTGACTCAAGGCATCTTCACCA | NM_181668.1 |
|  |  | Reverse | AAGAGCTCATCTCTGGGCTCACAT |  |
| FAM40A | hypothetical protein LOC511120 | Forward | TGGCTTCTCTTTGATTCCCAGGGT | NM_001075496.1 |
|  |  | Reverse | ACAAAGGAGCTGCAACCAACTGTG |  |
| S100A11 | S100 calcium binding protein A11 | Forward | ACAACAGCAAACTCTCCAAGGCTG | BC142378.1 |
|  |  | Reverse | ACCAGGGTCCTTCTGGTTCTTTGT |  |
| CD53 | CD53 molecule | Forward | TGCCATGTGCTGGAGAGACTTCTT | NM_001034232.2 |
|  |  | Reverse | TGGGAGGGAGAACAAAGACACCAA |  |
| DPYD | dihydropyrimidine dehydrogenase | Forward | TGCAACATCCGCTTTACGTTGTGG | NM_174041.2 |
|  |  | Reverse | GCATGGCAACAATTCTCCCACCTT |  |
| ELOVL4 | elongation of very long chain fatty acids -like 4 | Forward | TGCAGTCTCCACTGCCTACACTTT | NM_001099050.1 |
|  |  | Reverse | AACGCATCTGGAAAGGTTCTCGGT |  |
| CTSS | cathepsin S | Forward | ATGGGAATAAAGGCTGCAATGGCG | NM_001033615.1 |
|  |  | Reverse | TGGCACTTTCCATCCATGGCTTTG |  |
| C/EBPα | CCAAT/enhancer binding protein, alpha | Forword | AGAAGTCCGTGGACAAGAACAGCA | NM_176784.2 |
|  |  | Reverse | ATTGTCACTGGTCAGCTCCAGCA |  |
| PPARγ | peroxisome proliferator-activated receptor gamma | Forword | AGCCTCATGAAGAGCCTTCCAACT | NM_181024.2 |
|  |  | Reverse | TCCATAGTGGAACCCTGACGCTTT |  |
