# Supplementary material for: Characterization of Genes for Beef Marbling Based on Applying Gene Coexpression Network
Source: Int J Genomics. 2014 Jan 30;2014:708562. doi: 10.1155/2014/708562 (PMC3929194; doi:10.1155/2014/708562)
Supplement: Supplementary file 1 — Table S1 shows “Summary of microarray data sets”. Figure S1 shows “the distribution before and after normalization”. [file 708562.f1.zip › 708562.f1/mat.708562.v2.pdf]

(A)

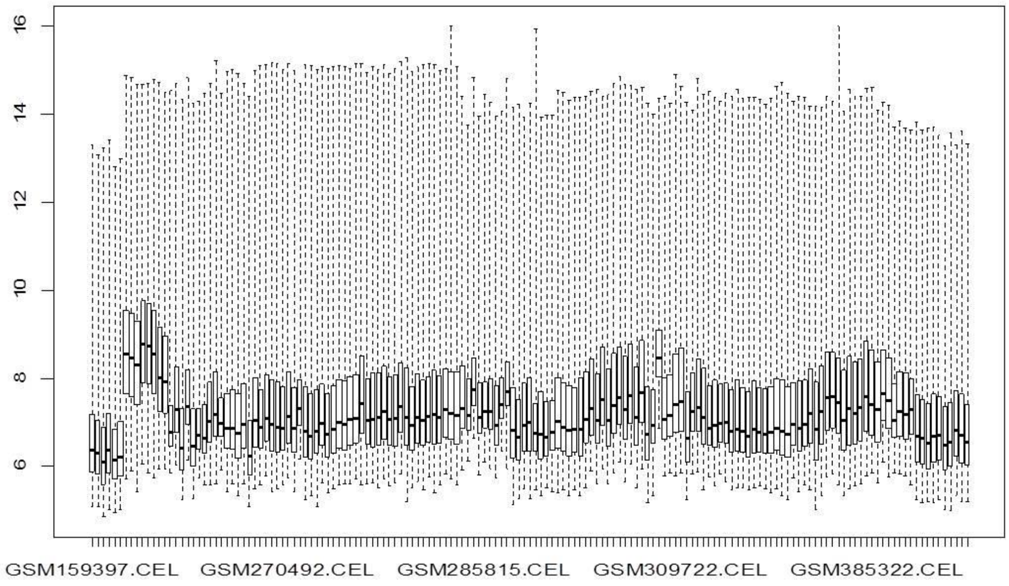
16
14
12
10
8
6
GSM159397.CEL GSM270492.CEL GSM285815.CEL GSM309722.CEL GSM385322.CEL

(B)

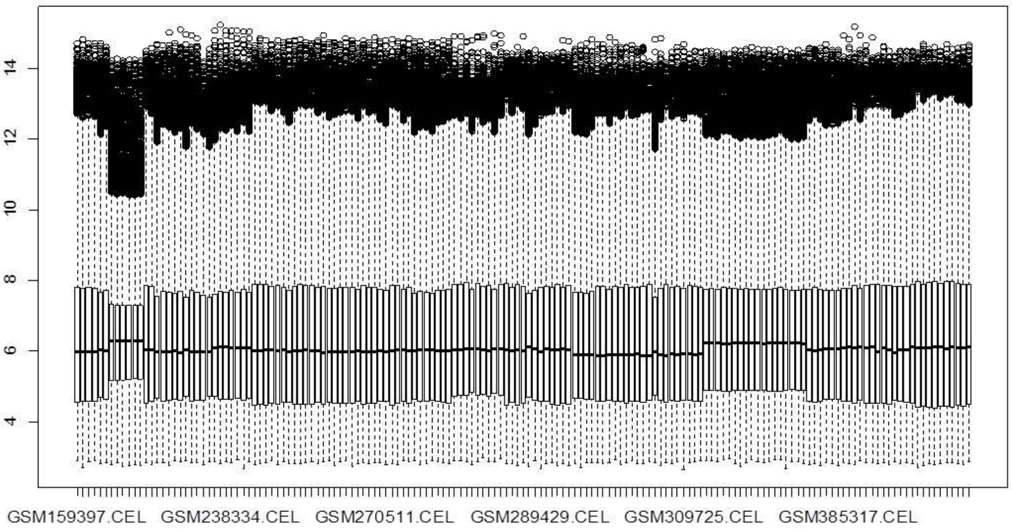
14
12
10
8
6
4
GSM159397.CEL GSM238334.CEL GSM270511.CEL GSM289429.CEL GSM309725.CEL GSM385317.CEL

**Supplementary Data 1. Primer sequences for real-time PCR**

| Gene symbol | Full name | | Primer sequence (5`-3`) | GenBank Acc. No. |
|---|---|---|---|---|
| MAEL | maelstrom homolog | Forward | ACACAAGGTGCAAGTGGCATGAAG | NM_001038193.1 |
| | | Reverse | ACATGAGCCTCTGTGAGCTGGATT | |
| HINT1 | histidine triad nucleotide binding protein 1 | Forward | TGGGCCTGAAGAAAGGCTATCGAA | NM_175812.2 |
| | | Reverse | TTCATCTGCCGACCTCCAAGAACA | |
| KIAA1712 | KIAA1712 | Forward | TTTGAGAGGAGCAGCATTCCCAGA | NM_001076833.1 |
| | | Reverse | CGTTTCCTACTTGGCAGGCAGATT | |
| TMEM60 | transmembrane protein 60 | Forward | AACTGGATGAGAAGGCACCTTGGA | NM_001076988.1 |
| | | Reverse | TGTGTGATCCATGTCGAGGGTCAA | |
| RHEBL1 | Ras homolog enriched in brain like 1 | Forward | AGCTGACTCAAGGCATCTTCACCA | NM_181668.1 |
| | | Reverse | AAGAGCTCATCTCTGGGCTCACAT | |
| FAM40A | hypothetical protein LOC511120 | Forward | TGGCTTCTCTTTGATTCCCAGGGT | NM_001075496.1 |
| | | Reverse | ACAAAGGAGCTGCAACCAACTGTG | |
| S100A11 | S100 calcium binding protein A11 | Forward | ACAACAGCAAACTCTCCAAGGCTG | BC142378.1 |

| | | | | |
|---|---|---|---|---|
| | | Reverse | ACCAGGGTCCTTCTGGTTCTTTGT | |
| CD53 | CD53 molecule | Forward | TGCCATGTGCTGGAGAGACTTCTT | NM_001034232.2 |
| | | Reverse | TGGGAGGGAGAACAAAGACACCAA | |
| DPYD | dihydropyrimidine dehydrogenase | Forward | TGCAACATCCGCTTTACGTTGTGG | NM_174041.2 |
| | | Reverse | GCATGGCAACAATTCTCCCACCTT | |
| ELOVL4 | elongation of very long chain fatty acids -like 4 | Forward | TGCAGTCTCCACTGCCTACACTTT | NM_001099050.1 |
| | | Reverse | AACGCATCTGGAAAGGTTCTCGGT | |
| CTSS | cathepsin S | Forward | ATGGGAATAAAGGCTGCAATGGCG | NM_001033615.1 |
| | | Reverse | TGGCACTTTCCATCCATGGCTTTG | |
| C/EBPα | CCAAT/enhancer binding protein, alpha | Forword | AGAAGTCCGTGGACAAGAACAGCA | NM_176784.2 |
| | | Reverse | ATTGTCACTGGTCAGCTCCAGCA | |
| PPARγ | peroxisome proliferator-activated receptor gamma | Forword | AGCCTCATGAAGAGCCTTCCAACT | NM_181024.2 |
| | | Reverse | TCCATAGTGGAACCCTGACGCTTT | |
